# Acupuncture Induces Time-Dependent Remodelling Brain Network on the Stable Somatosensory First-Ever Stroke Patients: Combining Diffusion Tensor and Functional MR Imaging

**DOI:** 10.1155/2014/740480

**Published:** 2014-07-02

**Authors:** Lijun Bai, Yin Tao, Dan Wang, Jing Wang, Chuanzhu Sun, Nongxiao Hao, Shangjie Chen, Lixing Lao

**Affiliations:** ^1^The Key Laboratory of Biomedical Information Engineering, Ministry of Education, Department of Biomedical Engineering, School of Life Science and Technology, Xi'an Jiaotong University, Xi'an 710049, China; ^2^Baoan Hospital, Southern Medical University, Shenzhen 518101, China; ^3^School of Chinese Medicine, The University of Hong Kong, 10 Sassoon Road, Pokfulam, Hong Kong; ^4^Center for Integrative Medicine, School of Medicine, University of Maryland, 520 W. Lombard Street, Baltimore, MD 21201, USA

## Abstract

Different treatment interventions induce distinct remodelling of network architecture of entire motor system. Acupuncture has been proved to be of a promising efficacy in motor recovery. However, it is still unclear whether the reorganization of motor-related brain network underlying acupuncture is related with time since stroke and severity of deficit at baseline. The aim of study was to characterize the relation between motor-related brain organization following acupuncture and white matter microstructural changes at an interval of two weeks. We demonstrated that acupuncture induced differential reorganization of motor-related network for stroke patients as time-lapse since stroke. At the baseline, acupuncture can induce the increased functional connectivity between the left primary motor cortex (M1) and the right M1, premotor cortex, supplementary motor area (SMA), thalamus, and cerebellum. After two-week recovery, the increased functional connectivity of the left M1 was more widely distributed and primarily located in the insula, cerebellum, basal ganglia, and SMA. Furthermore, a significant negative relation existed between the FA value in the left M1 at the baseline scanning and node centrality of this region following acupuncture for both baseline and two-week recovery. Our findings may shed a new insight on understanding the reorganization of motor-related theory underlying motor impairments after brain lesions in stroke patients.

## 1. Introduction

Stroke is responsible for increasingly high rates of mortality and disability worldwide [1]. Due to an aging population, dietary changes, and work-related stress, stroke morbidity is on the rise and the age at first occurrence is getting younger [2–4]. Despite considerable research efforts on multiple treatment modalities, there is still no single rehabilitation intervention demonstrated unequivocally to aid stroke recovery [5]. This reality drives people to search for other modalities of treatment in an attempt to further improve the outcome of stroke rehabilitation, such as acupuncture [5]. Acupuncture has been used in traditional Chinese medicine for more than 3000 years as a treatment for many diseases, and its use for poststroke rehabilitation in China is based on a large body of preclinical and clinical research [6]. As a therapeutic intervention acupuncture is also increasingly practiced in some western countries [7].

According to a conventional Western medical perspective, acupuncture has been described as increasing the pain threshold through needle activation of pain receptors by sending signals to the central nervous system to release opioid peptides [8]. In an analogous way, acupuncture may help in rehabilitation. A potential explanation is that analgesia achieved through acupuncture may relax muscles allowing for passive motion, an increased range of motion, and ultimately motor impairment rehabilitation [8]. It has also been suggested that adaptive changes in response to stroke involve neuronal reorganization and increased dendritic volume in the cortical layers and number of synapses in the contralateral hemisphere within 30 days after central nervous system injury [9]. A study indicates that Baihui (GV20-) based scalp acupuncture could improve infarct volume and neurological function score and exert potential neuroprotective role in experimental ischemic stroke [10]. In the acupuncture group, all the sensory, motor, and functional scores improved significantly during the examination period until 2 years after injury [11]. Scalp acupuncture combined with body acupuncture can evidently improve limb movement function and reduce the nerve function damage in stroke patients [12]. Brain signal activations during the same acupuncture were different between the healthy and the stroke patients, and the effects showed a correlation of different acupuncture points [13, 14]. Active acupuncture results in lateralization of functional cerebral response to the contralateral unaffected hemisphere in patients with unilateral stroke. This lateralisation may represent an effect of acupuncture in enhancing a compensatory process by redistributing functions into the intact cortex, particularly in the unaffected hemisphere [15].

Longitudinal studies of fMRI changes in stroke demonstrated that in the first few days after ischaemia the connectivity between the ipsilesional supplementary motor area and ventral premotor cortex with ipsilesional M1 was significantly reduced [13]. Coupling parameters between these areas were increased with recovery and predicted a better outcome 3–6 months later. Thus, a tight relation may exist between changes of motor system activity, premotor-M1 connectivity, and early recovery after stroke. It seemed that changes of interhemispheric connectivity within the motor-related brain network may largely depend on the time elapsed since stroke. In some patients with good motor recovery, the contralesional M1 seems to maintain a supportive effect on ipsilesional M1 activity [13]. These findings are also supported by transcranial magnetic stimulation, electroencephalography, and magnetoencephalography data in patients with good recovery; the contralesional hemisphere contributes to the degree of recovered function [14–16]. Therefore, treatments for improved motor performance may lead to a remodelling of the neural network architecture of the entire motor system towards a physiological state (i.e., stronger facilitation from the premotor cortex and less inhibition from the contralesional M1) [17].

Since there is no unique reorganization scheme with supportive or maladaptive effects of certain brain areas, there are substantial implications that various treatment interventions may lead to different reorganization of motor-related brain network which largely relied on the time elapsed since stroke. DTI have been shown to be sensitive to white matter (Al-Sadi, Newman et al.) damage, not only inside focal lesions but also in user-defined regions in the so-called normal appearing white matter (NAWM). New analysis techniques for DTI measures are now available that allow for hypothesis-free localization of damage and user-independent voxelwise analysis of DTI data. In the present study, we aimed to address the following hypotheses: (i) acupuncture stimuli may lead to specific brain responses in different time-period of recovery stroke at an interval of two weeks;,(ii) there may be a linkage between the brain responses to acupuncture and the brain microstructure DTI impairments in the individual stroke patients.

## 2. Materials and Methods

### 2.1. Subjects

A total of 9 patients (7 males, mean age: 57.7 ± 9.92 years), recruited from Beijing Dongzhimen Hospital, were diagnosed with right hemispheric striatocapsular infarction and stable ischemic stroke by MRI with unilateral upper-limb disability. The criteria for patient recruitment are listed as follows: (1) stable recovery stroke patients: >2 weeks and <12 weeks after the onset of stroke (first episode of stroke), (2) sufficient cognition to follow simple commands (Mini-Mental State Examination score) MMSE >21. Patients were excluded if they met any of the following criteria: (1) bilateral infarcts, (2) recurrent stroke, (3) any previous history of alcohol or drug abuse, (4) history of epilepsy or other neurological disease and psychiatric disorder, (5) serious cognitive deficits, comprehensive aphasia, and (6) other MRI contraindications (such as claustrophobia, etc.). The topographic distribution of the somatosensory deficit and the anatomic reconstruction of the brain lesions were shown in Table 1. Another 8 age-matched and sexually matched normal subjects (6 Males, mean age: 51.6 + 4.8 years) who were also recruited from Beijing Dongzhimen Hospital served as healthy controls. Each of them has normal neurological examination, no history of epilepsy or other neurological disease, psychiatric disorder, and other MRI contraindications (such as claustrophobia, etc.). All of the patients and the normal subjects are with right-hand dominance.

### 2.2. Clinical Assessments

Each patient underwent a series of clinical evaluations. Clinical outcomes measurements included the National Institute of Health Stroke Scale (NIHSS), Ashworth Scale for clinical measure of muscle spasticity, Brunnstrom for sequential motor recovery, Rankin Scale for stroke disability, Barthel Index of Activities of Daily Living, and Motricity Index. The anatomic reconstructions of the brain lesions are listed in Table 1. One patient had only taken part in the first scanning and the second clinical assessments and scanning.

### 2.3. fMRI Motor Task

**Figure 1:**
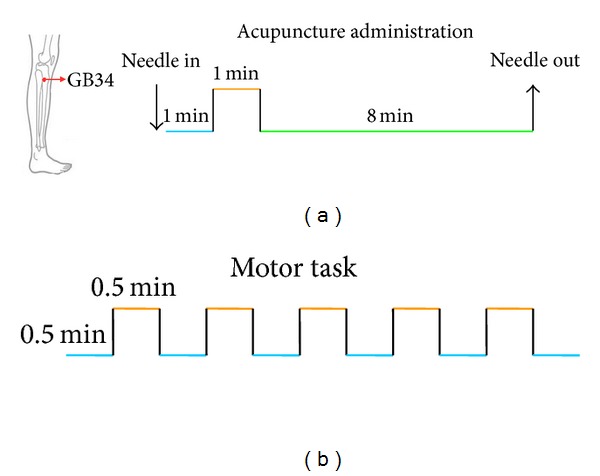
Acupuncture stimuli and motor task experimental paradigm.

During fMRI scanning, a simple finger movement was firstly served as stimulation for patients. A simple block design was performed in which 30-second baseline and 30-second stimulation alternated and lasted for 5 minutes and 30 seconds, with 10-second rest in the beginning. And the healthy subjects had the same MRI procedure as the patients (Figure 1).

### 2.4. Acupuncture Stimulation

Acupuncture stimulation employed the nonrepeated event-related design paradigm scanning, incorporating 1 min needle manipulation, preceded by 1 min rest epoch, and followed by 10 min rest scanning (without acupuncture manipulation) (Figure 1). Acupuncture was performed at acupoint GB34 on the left leg (located in the lateral aspect of the posterior knee). According to the TCM, the first choice acupoint for stroke is located at the scalp. Considering both limitation of fMRI scanning and classic use, we selected Yangming channel for Wei syndrome. GB34 is one of the most frequently used acupoints and proved to have various efficacies in the treatments of hemiplegia and rehabilitation for motor functional deficit/impairment after stroke. Acupuncture stimulation was delivered using a sterile disposable 38-gauge stainless steel acupuncture needle, 0.2 mm in diameter and 40 mm in length. The needle was inserted vertically to a depth of 2-3 cm, and the administration was delivered by a balanced “tonifying and reducing” technique. The stimulation consisted of rotating the needle clockwise and counterclockwise for 1 min at a rate of 60 times per min. The procedure was performed by the same experienced and licensed acupuncturist on all participants. Every subject endured twice acupuncture stimulation scanning at an interval of two weeks in order to test the differential brain response induced by acupuncture as the time-lapse in stroke recovery phrase.

### 2.5. Imaging Data Acquisition

The images were acquired on a 3T Siemens MRI Scanner. A custom-built head holder was used to prevent head movements. Thirty-two axial slices (FOV = 225 mm × 225 mm, matrix = 64 × 64, thickness = 3.5 mm) parallel to the AC-PC plane and covering the whole brain were obtained using a T2*-weighted single-shot, gradient-recalled echo planar imaging (EPI) sequence (TR = 2000 ms, TE = 30 ms, flip angle = 90°). Prior to the functional run, high resolution structural information on each subject was also acquired using 3D MRI sequences with a voxel size of 1 mm^3^ for anatomical localization (TR = 1.9 s, TE = 2.52 ms, matrix = 256 × 256, FOV = 250 mm × 250 mm, flip angle = 9°, and slice thickness = 1 mm).

### 2.6. Functional Data Analysis

All preprocessing steps were carried out using statistical parametric mapping (SPM5, http://www.fil.ion.ucl.ac.uk/spm/). The images were first slice-timed and then realigned to correct for head motions (none of the subjects had head movements exceeding 1 mm on any axis and head rotation greater than one degree). The image data was further processed with spatial normalization based on the MNI space and resampled at 2 mm × 2 mm × 2 mm. Finally, the functional images were spatially smoothed with a 6 mm full-width-at-half maximum (FWHM) Gaussian kernel. The statistics were color-coded and mapped in the Talairach space. A finite-impulse response band-pass filter was applied to the dataset used for functional connectivity analyses in order to remove the frequency out of the 0.01–0.08 Hz signals.

For motor task, statistical analysis was performed in two steps. First, a single subject fixed effects model was used. The difference between the motor condition and the resting was estimated at each voxel by using the general linear model (GLM) and the parameter estimates for the covariate resulting from the least mean square fit of the model to the data were calculated. In second-level analysis, the obtained individual *t*-maps were used in “random effect” group analysis framework by one-sample *t*-test for different groups. The statistical threshold was set at *P* < 0.05 (corrected for multiple comparisons). We selected the left primary motor cortex (M1) as the seed region for further analysis.

### 2.7. M1-Seeded Brain Network Induced by Acupuncture Effect

Since the effect of acupuncture can sustain beyond the needle manipulation period, it will modulate the moment-to-moment processes relevant to the poststimulus resting brain when there is no longer external stimulus. In the present study, we aimed to investigate the M1-related resting brain network induced by acupuncture stimulus. The peak voxel and its 6 nearest neighbors were defined as the ROI.

For each subject, the “seeding” time courses of the left M1 were, respectively, cross-correlated with all low-pass filtered voxels to generate functional connectivity maps within each of the three conditions. The resulting correlation coefficient *r*-maps were normalized and corrected to roughly standard normal distributions using the methods previously described [18, 19]. The normality of the distribution was then tested using Kurtosis tests (*P* < 0.05). The *z*-maps of each individual were entered into one-sample *t*-tests, respectively, to determine whether the group data was significantly different from zero.

### 2.8. TBSS Analysis

We use FSL4.1.2 (FSL 4.1.2; http://www.fmrib.ox.ac.uk/fsl/) to calculate the FA value. To calculate FA value for each voxel, we do the following: (1) eddy current correction using weighted data, (2) creating brain mask using unweighted data after running standard brain extraction using bet 2, (3) DTIFIT reconstructing diffusion tensor with weighted data. Then, a voxel-wise statistical analysis of the FA data is carried out using tract-based spatial statistics (TBSS) V1.2 part of FSL. All subjects' masked FA data were then aligned into a common space using the nonlinear registration tool FNIRT, which uses a *b*-spline representation of the registration warp field. Next, a mean FA image was created and thinned to create a mean FA skeleton, which represents the centers of all tracts the group has in common. Each subject's aligned FA data were then projected onto this skeleton. All analyses were masked to only display regions with FA values of >0.2 and <0.8 as an additional procedure to avoid examination of regions that are likely comprised of multiple tissue types or fiber orientations. Whiter matter FA value changes were assessed using permutation-based nonparametric testing with 5000 random permutations. We only displayed changes with a *P* < 0.005 and the cluster of voxel >5.

### 2.9. Correlation Analysis

Correlations among global DTI measures and clinical scores (NIHSS, Ashworth Scale for clinical measure of muscle spasticity, Brunnstrom for sequential motor recovery, Rankin Scale for stroke disability, and Motricity Index) were analyzed by univariate analysis (Pearson's correlation coefficient) after correction for age, and results corrected for multiple comparisons were needed.

## 3. Results

### 3.1. Results from TBSS Analysis

**Figure 2:**
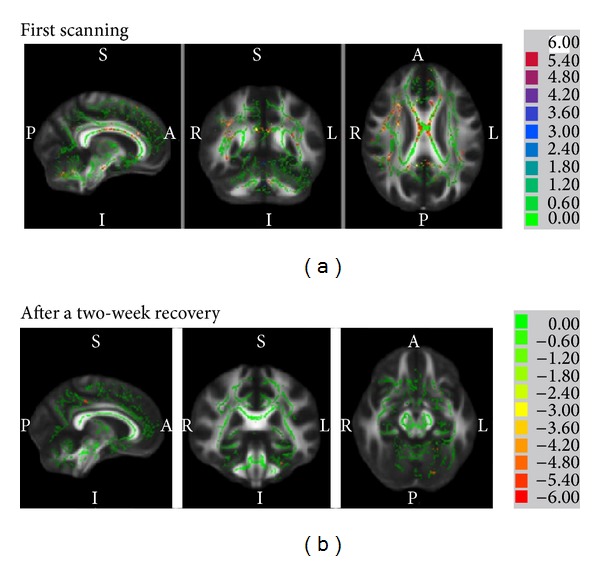
FA changes from TBSS analysis for before and after two-week recovery.

Our TBSS results revealed the decreased FA value of the cingulate gyrus, basal ganglia, corpus callosum, precuneus, inferior parietal lobule, left primary motor cortex, and insula in stroke patients compared with healthy controls (*P* < 0.05, Figure 2). The regions of increased FA value were primarily located in the cerebellum in stroke patients compared to control. After two-week recovery, the FA values in the insula, supplementary motor area, postcentral gyrus, parahippocampal gyrus, and cerebellum (BA27) were increased while the FA value in the cingulate gyrus and temporal lobe was decreased.

### 3.2. Correlation between Diffusion Parameters (FA) and Clinical Scores

**Figure 3:**
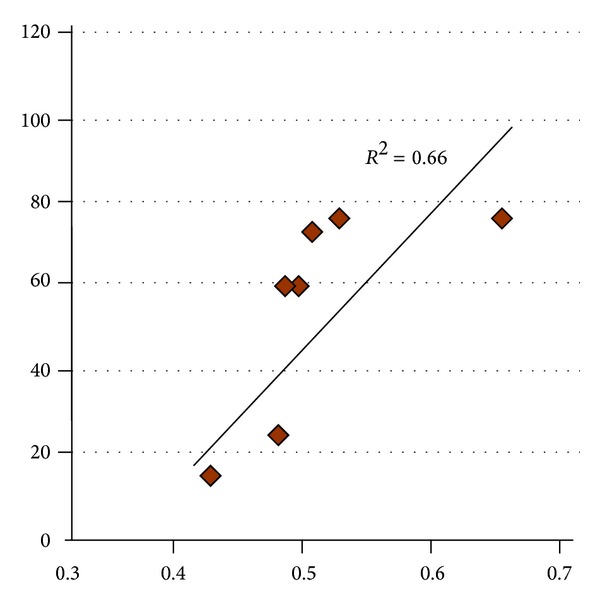
Positive correlation between the FA value in the left M1 and Motricity Index (*r* = 0.66, *P* < 0.05).

We found a positive correlation between the FA value of the M1 in stroke patients and the Motricity Index (*r* = 0.8124, *P* = 0.014, shown in Figure 3). Other clinical scores showed no significant relation with the FA values.

### 3.3. Acupuncture Stimuli for Stroke Patients

**Figure 4:**
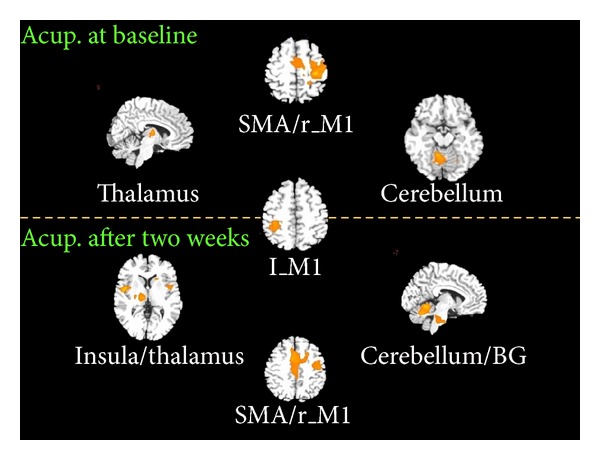
The left M1-anchorned brain network for both acupuncture at the baseline and two-week recovery (*P* < 0.001, FDR corrected). M1: primary motor cortex; SMA: supplementary motor area; BG: basal ganglia; l: left, r: right.

For the first acupuncture stimuli, acupuncture induced the increased functional connectivity of the left M1 with the right M1, premotor cortex, supplementary motor area, thalamus, and cerebellum. After two weeks of recovery, the increased functional connectivity of the left M1 was more widely distributed and primarily located in the insula, cerebellum, basal ganglia, and supplementary motor area while decreased functional connectivity was particularly located in the right M1 compared to that of the first scanning (Figure 4).

We also find a significant negative relation between the FA value in the left M1 at the baseline scanning and node centrality of the left M1 during the post-acupuncture resting network for both baseline and two-week recovery (*r* = 0.514, *P* < 0.05 for baseline; *r* = 0.647, *P* < 0.05 for two-week recovery).

## 4. Discussion

Our findings demonstrated that acupuncture may induce differential reorganization of motor-related network for stroke patients. The reorganization of motor-related network may be partly associated with the microstructure impairments of white matter revealed by DTI TBSS analysis. Acupuncture may provide a potential treatment for stroke motor recovery and more importantly give a new insight into the reorganization of motor-related theory underlying motor impairment after brain lesions for stroke patients. However, these inferences are preliminary and need to be systematically tested in future studies.

Motor function after stroke critically depends on corticospinal tract (CST) integrity [20]. The primary motor cortex has been shown to help encode the force of a muscle contraction using information that is sent back by the peripheral nervous system. The decreased FA in stroke patients in primary motor cortex is related to the upper-limb disability. Similar results could be seen in some resent studies [21]. The microstructure of primary motor cortex becomes intact which can improve the upper-limb ability. An extensive literature suggests that the contralesional cortex plays a role in recovery from a CNS lesion, particularly in humans [22]. Using different techniques, it has been demonstrated that the contralesional primary motor cortex exerts a persistent inhibitory drive over the ipsilesional primary motor cortex in the process of generation of voluntary movements by the paretic hand [23–26].

Whereas the initial increase of contralesional activity might be a passive phenomenon that is not related to function, in some cases, this increased activity persists long after the lesion [27]. Motor recovery can be mediated by increased activation of premotor cortical areas of both cerebral hemispheres [22].

The decreased FA value of the insula was found in stroke patients compared to healthy control at the baseline. Lesion and functional imaging studies in humans suggested that the insula and the peri-insular region also may be part of the human vestibular system [18, 19, 28–30] and may be involved in language and articulation processes in the left hemisphere [31, 32] and in processes of spatial exploration and orientation in the right hemisphere [33, 34]. One study observed decreased activity of the right posterior insula with a decreasing feeling of controlling the movement [35]. The destroyed microstructure of the insula can lead to the stroke patient's upper-limb disability. After a two-week recovery, the FA value of the insula was increased, which may be associated with improved motor. The integrity of insula microstructure can be used to evaluate the level of recovery. The insula plays an important role in recovery in stroke patients. The FA value in the cingulate cortex decreased compared to control which is consistent with previous studies [36]. One study showed that the posterior cingulate cortex is typically discussed as having a unitary function because of a common pattern of relative deactivation observed during attentionally demanding tasks [37]. It is a key node in the default mode network (DMN) and shows increased activity when individuals are in rest state and decreased activity when individuals are at task. The cingulate cortex loss will lead to completing the function of DMN. The DMN cannot decrease activity when the stroke patients do not have the ability to move their upper-limb.

Abundant neuroimaging studies have indicated that the exact functional reorganization of brain regions during recovery seems to be complex. Most likely, time since stroke, severity of deficit at baseline, lesion size, location, and other biological factors (e.g., age of the patient) all contribute to interindividual differences. More importantly, both impairment and compensatory processing of brain after stroke are highly relevant with respect to the development of new treatment approaches. In other words, different treatment interventions may induce distinct remodelling of the neural network architecture of the entire motor system towards a more physiological state. In the present study, we provide a possibility that the functional brain connectivity anchored by the left M1 following acupuncture stimuli may be partly related to the impairments of white matter microstructures in stroke patients. Particularly, acupuncture effect on stroke patients may largely be dependent on the time-lapse till stroke lesion. At the baseline, acupuncture can induce the functional connectivity between the left M1 and right M1, premotor cortex, supplementary motor area, thalamus, and cerebellum. By contrast, after a two-week recovery, more wide range of brain network was induced by acupuncture stimuli.

There are some limitations in the present study. First, we only include small sample of stroke patients while both lesion size and location were relatively consistent.

The inference needs to be systematically tested in larger sample in future studies. Second, we do not measure the degree of the stroke patients' recovery. In further study, we will expand the scope of the study population, conduct longitudinal observation on the basis of the study, and analyze the relationship between the different degree impairment and the degree of recovery after acupuncture.

## 5. Conclusion

The present study provided a clue to a linkage between the brain responses to acupuncture and microstructure DTI impairments in the individual stroke patients. Time since stroke and severity of deficit at baseline may partly contribute to interindividual differences in functional connectivity within the motor-related brain network induced acupuncture. Different treatment interventions may lead to distinct remodelling of the neural network architecture of the entire motor system towards a more physiological state.

## Figures and Tables

**Table 1:** Clinical and demographic data.

Patient number	1	2	3	4	5	6	7	8	9
Age (years)	56	64	57	68	57	37	58	71	52

Gender	F	M	M	M	F	M	M	M	M

Localization of infarct	BG	IC	IC	CR	IC	IC	IC	IC	BG

Motricity Index	0	60	14	72	23	60	34	76	76
	11	64	14	72	23	60	34	76	—

Rankin Scale	4	1	2	2	4	2	3	2	2
	4	1	2	1	4	2	3	1	—

Barthel Index	35	95	60	90	60	85	65	90	85
	40	95	65	85	60	85	75	90	—

NIHSS	14	3	9	5	8	7	7	3	5
	8	1	9	2	8	7	7	2	—

MMSE	22	30	27	29	22	30	30	24	30
	23	30	30	28	24	30	30	27	—

Brunnstrom	I	IV	II	II	I	V	II	V	II
	I	IV	II	III	I	V	II	V	—

Asworth	0	1	1	0	0	2	2	0	0
	0	1	0	1	0	2	2	0	—

BG: basal ganglia; IC: internal capsule; CR: corona radiate; NIHSS: National Institute of Health Stroke Scale; MMSE: Mini-Mental State Examination.
